# Kainar Syndrome: History of the First Epidemiological Case-control Study of the Effect of Radiation and Malnutrition

**DOI:** 10.5195/cajgh.2015.221

**Published:** 2015-01-23

**Authors:** Aidar Atchabarov

**Affiliations:** Atchabarov Institute for Basic and Applied Biomedical Research, Asfeniyarov National Medical University, Almaty, Kazakhstan

Kainar Syndrome was originally described in the late 1950s as a health condition associated with increased bleeding, hair loss, fainting, and fatigue.^1^ The name “Kainar” came from the village in Kazakhstan where most of the affected patients resided. Initial explorations of the etiology of Kainar Syndrome identified radiation exposure and insufficient levels of vitamin C as risk factors for the syndrome.

An extensive study of the syndrome was conducted by the National Academy of Sciences of Kazakh SSR from 1957 to 1960,^1^,^2^ which established the presence of harmful effects of nuclear tests in Semipalatinsk Nuclear Test Site (SNTS) on human and animal health.^3^ However, not all results were published, as authorities did not believe that such a condition existed. Once the results of this research became known to the leaders of Kazakhstan and the former Soviet Union, the Military-Industrial Complex grew more concerned about the health effects of nuclear testing, and declared a moratorium on nuclear testing. Since 1961, dangerous ground-level tests ceased; in 1962, testing was switched to high-altitude tests; and in 1963, only underground tests were allowed. On August 5, 1963, the Limited Test Ban Treaty was signed, banning nuclear weapon testing in the atmosphere, outer space, and underwater.^4^,^5^

The Institute of Regional Pathology in Alma-Ata, the former capital of Kazakhstan (currently, Almaty), was founded due to the need to better understand the effects of penetrating radiation. This need arose due to the emergence of a new understanding that the early appearance of radiation sickness symptoms mimicked various other pathologies (e.g. infection, intoxication, burns, etc.). Previous understanding of radiation sickness assumed that radiation sickness primarily manifested itself in genetic mutations at the cellular, tissue, and organ levels. The goal of this research program was to develop in-depth comprehensive studies of health outcomes impacted by radiation effects.

## A pilot study^6^

A group of researchers led by Dr. Bahia Atchabarov, a renowned Kazakh scientist, recruited 3,564 cases (individuals who lived in radiation exposed areas of the former Semipalatinsk region) and 2,028 controls (individuals in areas without contamination) and examined their health status. Cases were recruited from three radiation exposed areas of the former Semipalatinsk region (Abai, Beskaragai, and Shubartausky), and controls were recruited from four areas without contamination Zhezkazgan (Ulutausky district), Karaganda (Kounrad and Kuvsky areas), and Pavlodar (Bayanaul area) regions. Participants were matched based on key baseline health characteristics. In order to ensure the reliability of findings on health effects of radiation exposure, animal populations (farm animals) were also studied in addition to the human participants.

To confirm the clinical data identified in humans and to clarify the severity of radiation influence on health, the health status of farm animals (1,257 cows and 1,000 sheep) from the Abai (case area) district and four control areas (800 cows and 605 sheep) were assessed.

### Radiation in animals

It was concluded that animals examined in the Abai district suffered from hypochromic anemia, accelerated erythrocyte sedimentation rate, erythrocyte dysfunction, leucopenia, eosinophilia, stab and segmented neutrophilia, leukocytopenia, abnormality of redox processes of cellular respiration, decreased concentration of vitamin C in the blood, and abnormal liver function. Extensive pathological changes in organs and tissues of animals in Abai district were similar for all animals examined. The overall pattern of health deterioration was characterized by degenerative changes and sclerotic processes in parenchymal organs, tissue atrophy and hyperplasia of the respiratory and digestive tracts, pre-cancerous lesions in the lungs, degenerative changes in cortical neurons and the brain stem, a decrease in the islet apparatus of the pancreas, inhibition of germinal elements in the ovaries, and distinct changes in the thyroid gland. Pronounced changes were also identified in the mucosa of the upper respiratory tract. There were areas of acute thinning and destruction of the epithelial lining, formation of dense cellular infiltrates beneath the epithelium, acute proliferation of smooth muscle, and abnormal intestinal wall elements, with an increasing number of nuclei in which amniotic division occurs. There were also pronounced inflammatory-degenerative changes in the thyroid gland.

Pathological changes in farm animals were more pronounced than changes in human participants. It was hypothesized that this may be due to the fact that animals were held in the fields during environmental radiation exposure without protective cover. Animals also consumed forage contaminated with radioactive particles, and were in constant contact with the surface soil contaminated with radioactive fallout.

### Radiation in participants

Human research participants examined in the Semipalatinsk region (especially among the population of the Abai district) had a higher number of clinical symptoms when compared to participants from control areas. These symptoms included: hemorrhages in the mucous membranes of the upper respiratory tract, mouth, and genitals, degenerative changes in the mucous membranes (e.g. erosion, leukoplakia, hyper pigmentation, and telangiectasia), abnormal changes in peripheral blood (e.g. leukopenia, lymphopenia, thrombocytopenia, lymphocytosis, anemia, and white blood cell stimulation), cataracts diagnosed at young age, increased fragility of blood vessels and capillaroscopic changes, changes in menstrual and ovarian cycles, abnormal liver function, atrophic rhinitis and pharyngitis, gingivitis, pathologies of the gastrointestinal tract (e.g. gastritis), skin changes - particularly on skin surfaces not protected by clothing (e.g. hyperkeratosis and hyper pigmentation), nail dystrophy, asthenic and wasting syndromes, and hypotension.

The frequency of detection of pathological changes in individuals who lived in areas with radioactive contamination was significantly higher than those from the control areas. Pathological changes identified in human participants and animal subjects were not specifically attributed to any well-known common diseases characterized by inflammatory, degenerative, or sclerotic changes in the organs.

In the evaluation of clinical samples, all participants were classified into four groups based on the number and severity of symptoms: A, B, C, and D. Group A included individuals with the highest number of symptoms, many of which could be attributed to the nature and severity of the symptoms of chronic radiation sickness. Group B had the second greatest number of symptoms, with greater than three clinical symptoms with the disease. Group C consisted of individuals who had two or three clinical symptoms of the disease, which (although they could occur in patients with chronic radiation sickness) more often were symptoms of well-known chronic somatic and infectious diseases. Group D consisted of healthy individuals without any clinical symptoms of the disease or in the presence of one or two clinical symptoms within the normal range for residents of those areas.

### Introduction of Kainar Syndrome

Later, the symptoms of the groups that exhibited clinical manifestations (A–C) were named “Kainar Syndrome,” after the village where these clinical manifestations were most prevalent. Kainar Syndrome was very common among the symptomatic population in the surveyed regions, with a prevalence ranging from 48.2% to 62.6% in control areas, and ranging from 72.8% to 96.2% in areas with radiation contamination. In areas with radiation contamination and an increase in the disease frequency, the incidence of severe forms of disease also increased. Kainar Syndrome was divided into two groups based on the number of clinical symptoms and their severity: strongly pronounced rate - “Kainar A” (or “Major Kainar”) and weakly pronounced rate - “Kainar C” (or “Minor Kainar”). Major Kainar includes the clinical manifestations of groups A and B, whereas Minor Kainar includes the clinical manifestations of the group C.

Major Kainar encompasses the largest number of clearly defined clinical manifestations: vascular changes, hematopoietic system changes, functional changes in the nervous system (i.e. indicating the presence of wasting syndrome), and changes more specific to penetrating radiation exposure (e.g. changes in the eye lens at a young age and changes in exposed skin). Minor Kainar encompasses individuals with 2–3 clinical manifestations of mild severity, which have similar characteristics for chronic radiation sickness. Individuals with Minor Kainar also develop infectious diseases or toxicities typically associated with age or with hypovitaminosis, in addition to mild radiation exposure.

The prevalence of Major Kainar in the contaminated areas was 3 to 5 times greater than that of the control areas. This demonstrates the potential role of radiation in the development of this disease. The next question is “What is the cause, mechanism, and etiology of Kainar Syndrome, particularly Minor Kainar, which is found even in populations in control areas?”

Previous studies have found that lack of essential vitamins, such vitamins C, B, and A, are associated with an increased risk of developing Kainar Syndrome.^1^ Based on the results of a previously published preliminary study,^6^ hypovitaminosis of vitamin C was associated with the development of Kainar Syndrome among the examined population in control areas. There was also a synergistic effect of radiation and hypovitaminosis of vitamin C in people residing in contaminated regions. According to the data, hypovitaminosis of vitamin C was identified as a very widespread condition among the examined population of Central Kazakhstan. Chronic radiation sickness was found to be aggravated by hypovitaminosis of vitamin C.^6^ However, results were preliminary, and further studies are needed to examine the etiology of Kainar Syndrome in Central Asia.

In conclusion, analysis of case-control data collected between 1957 and 1959 led to the assumption that pathological changes in humans and animals are directly related to radiation exposure. Kainar Syndrome was also found not to be specific for people living in areas with radioactive contamination, but is also found in people from control areas. While Kainar Syndrome, overall, has received significant attention in the literature, Minor Kainar Syndrome remains an underpublished phenomenon. Therefore, future research is needed to further investigate Kainar Syndrome in the geographic areas impacted by radiation exposure.
